# The Value of Morphometric Measurements in Risk Assessment for Donor-Site Complications after Microsurgical Breast Reconstruction

**DOI:** 10.3390/jcm9082645

**Published:** 2020-08-14

**Authors:** Muriel O. Meyer, Tristan M. Handschin, Daniel T. Boll, Frédérique Chammartin, Dirk J. Schaefer, Martin D. Haug, Elisabeth A. Kappos

**Affiliations:** 1 Department of Plastic, Reconstructive, Aesthetic and Hand Surgery, University Hospital of Basel, 4031 Basel, Switzerland; muriel.meyer@stud.unibas.ch (M.O.M.); tristan.handschin@stud.unibas.ch (T.M.H.); dirk.schaefer@usb.ch (D.J.S.); martin.haug@usb.ch (M.D.H.); 2 Faculty of Medicine, University of Basel, 4051 Basel, Switzerland; 3 Department of Radiology and Nuclear Medicine Clinic, University Hospital of Basel, 4031 Basel, Switzerland; daniel.boll@usb.ch; 4 Basel Institute for Clinical Epidemiology and Biostatistics, Department of Clinical Research, University Hospital and University of Basel, 4031 Basel, Switzerland; frederiquesophie.chammartin-basnet@usb.ch; 5 Breast Center, University Hospital of Basel, 4031 Basel, Switzerland

**Keywords:** breast reconstruction, autologous reconstruction, deep inferior epigastric perforator flap, muscle-sparing transverse rectus abdominis myocutaneus flap, donor-site complications, risk assessment, morphometric measurements

## Abstract

Microsurgical abdominally-based reconstruction is considered the gold standard in autologous breast reconstruction. Despite refined surgical procedures, donor-site complications still occur, reducing patient satisfaction and quality of life. Recent work has outlined the potential of morphometric measurements in risk assessment for postoperative hernia development. With rising demand for personalised treatment, the goal of this study was to investigate their potential in risk assessment for any donor site complication. In this retrospective cohort study, 90 patients were included who each received microsurgical breast reconstruction at the hands of one surgeon between January 2015 and May 2017. Donor-site complications formed the primary outcome and were classified according to Clavien–Dindo. Morphometric measurements were taken on a routinely performed computed tomographic angiogram. Complications occurred in 13 of the 90 (14.4%) cases studied. All patients who developed any type of postoperative donor site complication had a history of abdominal surgery. The risk of postoperative complications increased by 3% with every square centimetre of omental fat tissue (OR 1.03, 95% CI 1.00–1.06, and *p*-value = 0.022). Morphometric measurements provide valuable information in risk assessment for donor-site complications in abdominally-based breast reconstruction. They may help identify personalised reconstructive options for maximal postoperative patient satisfaction and quality of life.

## 1. Introduction

Breast cancer is the most common form of cancer in women worldwide [1]. Approximately one third of women diagnosed with breast cancer are still advised to undergo uni- or bilateral mastectomy [2]. Nowadays, more women than ever are surviving breast cancer, and various studies have proven that post-mastectomy breast reconstruction brings quality of life benefits to many of the cancer survivors [3,4,5,6].

A general advantage of reconstruction with autologous tissue is the natural cosmetic result with respect to texture, consistency, and adaptation to body weight fluctuations—even over longer periods of time. A disadvantage of autologous reconstruction compared to implant reconstruction is the possibility of donor-site complications, which may lead to additional patient morbidity [7,8,9,10].

Refinements in surgical techniques over the last decades have led to the development of perforator flaps with as little muscle and/or fascial sacrifice as the individual patient’s anatomy allows. This has reduced donor site morbidity, and complications have become rarer [11]. Nevertheless, donor-site complications like hernias, abdominal bulging, seromas, delayed wound healing, and infections can still occur, with corresponding implications for patient satisfaction and quality of life.

The latest scientific knowledge suggests that the thus far mentioned complications occur due to a multitude of causes associated with various patient-specific traits [10,12] and objective morphology-based parameters [13,14]. Numerous recently-published studies have shown the potential of objective morphometric measurements in risk assessment for different types of surgery [13,14,15,16,17].

Since breast cancer survival rates are high [18] and breast reconstruction is a purely elective surgery, it is particularly important to aim for the highest possible patient satisfaction by reducing donor-site complications.

Preoperative patient selection for surgery using a simple and readily available imaging method could provide great added value to the overall decision-making process when selecting personalised reconstructive treatment options. Such a method is not yet available as a clinical standard.

In clinical practice, a computed tomographic angiogram (CTA) is often part of the routine preoperative workup for perforator vessel assessment. As such, it is widely available and could hence easily be used in the future as a risk stratification tool before surgery. Therefore, we endeavoured to investigate the potential of preoperative CTA-derived morphometric measurements in risk assessment for donor-site complications.

## 2. Methods

### 2.1. Surgical Procedure

Deep inferior epigastric perforator (DIEP) flaps were the first choice for all microsurgical abdominally-based breast reconstructions. All DIEP flaps were harvested with no muscle and with minimal fascia and, whenever possible, with preserved motor innervation of the rectus abdominis muscle.

When using a muscle-sparing transverse rectus abdominis myocutaneus (ms-TRAM) flap, care was taken during flap elevation to ensure that the medial and lateral muscle sections were preserved to the maximum. Closure of the donor site was achieved by reattaching the anterior rectus fascia with a running 0-polypropylene suture (Prolene, Ethicon, Inc., Somerville, NJ, USA). Minimal fascial incision was made in every case, the extent of which depended on perforator location. Fascial incision was kept at 5 centimetre max. This was not correlated with previous abdominal surgery, as most patients had undergone caesarean section or laparotomy and not unilateral abdominal surgery. Preoperative rectus diastasis was repaired, whenever intraoperatively necessary, by a duplicating running suture, using the same 0-polypropylene stich. No mesh was used in any of the patients included in the study.

### 2.2. Computed Tomographic Angiogram Measurements

All patients who received abdominally-based breast reconstruction had also been routinely subjected to a preoperative CTA for perforator vessel mapping. These patients underwent four-phase dynamic CT examinations: unenhanced, arterial phase, portal venous phase, and 3-min delayed phase. The CT examinations were performed with a 128-slice CT (SOMATOM Definition Edge, Siemens Healthineers, Erlangen, Germany) or a 256-slice-image (SOMATOM Definition Flash, Siemens Healthineers, Erlangen, Germany) scanner system. Following the unenhanced scan, 1.2 mL/kg of 370 mg I/mL iopromide (Ultravist^®^ 370, Bayer Pharma, Leverkusen, Germany) was injected intravenously via a power injector (Ulrich Medical, Ulm, Germany). Using the bolus tracking technique, anteroposterior images were taken 18 s after achieving a 100 HU improvement in the descending aorta at the level of the celiac trunk.

**Figure 1:**
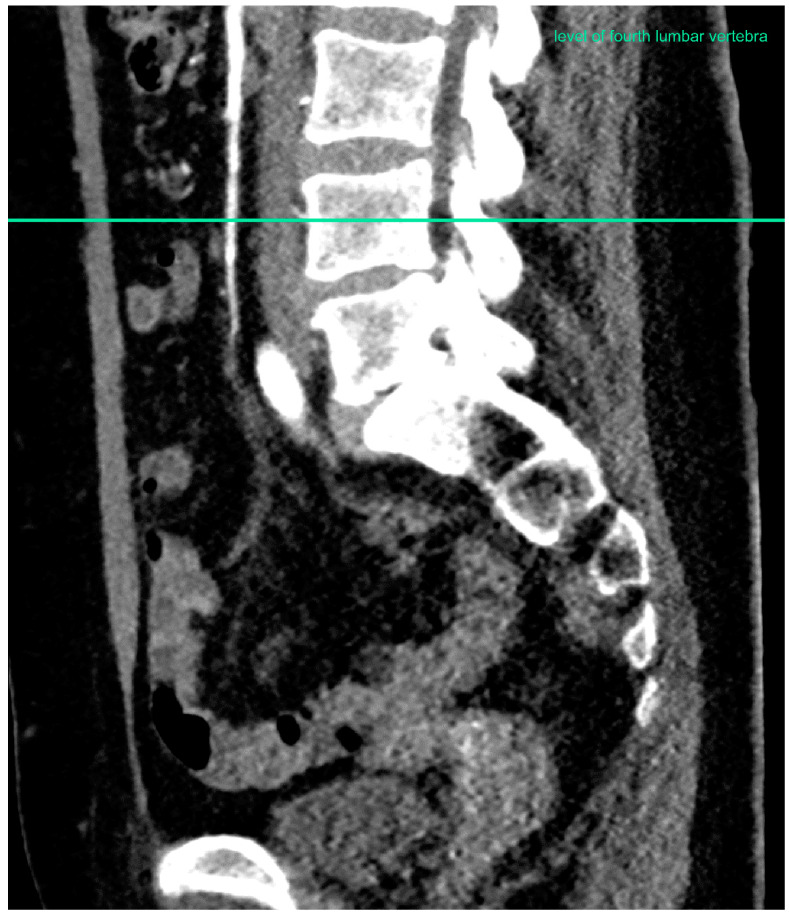
Level of fourth lumbar vertebra.

**Figure 2:**
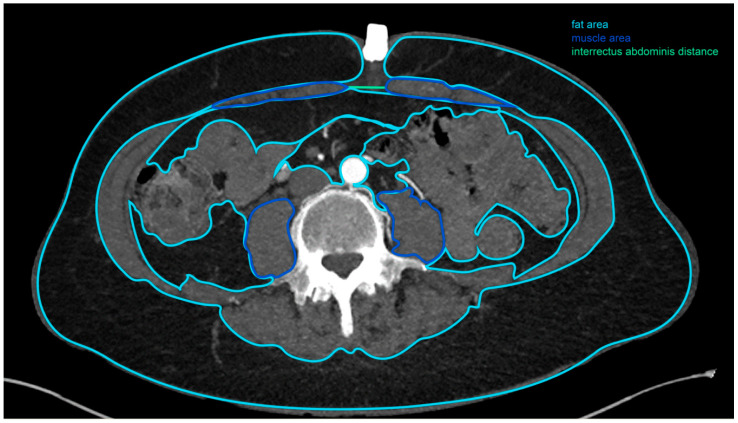
Computed tomographic angiogram (CTA) measurements.

All morphometric measurements were taken at the level of the fourth lumbar vertebra by one person (Figure 1). The image-based evaluation of each dataset was conducted using a commercially available post-processing software application (syngo. via VB30, Siemens Healthineers, Erlangen, Germany). In each patient, the following measurements were taken: interrectus abdominis distance, area and density of the right and left rectus muscle, area and density of the right and left psoas muscle, area and density of the subcutaneous, intra-abdominal and retroperitoneal fat tissue, as well as the maximal abdominal diameter, measured in the frontal and sagittal plane (Figure 2).

### 2.3. Patients

This study, which was conducted in accordance with the ethical principles of the Declaration of Helsinki, included all patients who underwent microsurgical breast reconstruction using abdominal tissue performed by one lead surgeon at one institution between January 2015 and May 2017 and who had received a preoperative CTA. In all patients included, the postoperative course was examined and the postoperative donor-site complications were classified into severity levels (Table 1). The standard follow-up consisted of daily clinical check-ups during the hospital stay and subsequent outpatient consultations 2 weeks, 6 weeks 3 months, 6 months, and one year after surgery. In the event of any complications at donor or recipient sites, patients were instructed to promptly contact their treating plastic surgeon. In this study, the duration of follow-up was between 19 and 48 months. The patients were allowed to resume full physical activity, including the unrestricted use of their core muscles, 3 months after surgery. With our minimum follow-up of 19 months, late-onset donor-site complications like hernias/bulges would have been detected as well.

To assess the donor-site complications, we chose the Clavien–Dindo classification. It is based on the therapeutic management of postoperative complications, and is the most frequently cited classification of surgical complications. Each grade is defined by a set group of possible medications and interventions that may be used to address a given postoperative complication. Thus, each and every postoperative complication can be assigned to the proper grade depending on the specific treatment regimen it necessitated. Through this objective, reproducible, and prevalent evaluation of surgical outcomes, this classification is a compelling tool for quality assessment in general surgery worldwide and enables inter-study comparability [19,20,21,22]. We appreciate and support the growing use of the Clavien–Dindo classification in the literature on plastic surgery [19,23,24,25], and which results in much facilitated comparison of surgical outcomes between surgeons, centres, and therapies.

Individual patient characteristics identified as potential risk factors in the literature were extracted from electronic patient charts. In particular, we considered obesity (yes/no), smoking habits (yes/no), diabetes mellitus (yes/no), age at surgery, type of surgical procedure (DIEP: yes/no), history of abdominal surgery (yes/no), and history of chemotherapy (yes/no). Obesity was defined as a body mass index (BMI) above 25 kg/m^2^ at the time of surgery. The distinction between the two surgical techniques used, DIEP (yes) and ms-TRAM (no) flaps, was recorded in the subgroup “type of surgical procedures”. Included in the subgroup “history of abdominal surgery” were caesarean sections, appendectomies, longitudinal laparotomies, abdominal hysterectomies, cholecystectomies, cyst enucleations, removal of haematomas, and one case of abdominal surgery in which the original indication was unclear. Some of the included patients underwent more than one abdominal surgery before receiving abdominally-based breast reconstruction. The subgroup “history of chemotherapy” included all patients who had received chemotherapy at any point in time prior to surgery.

### 2.4. Statistical Analysis

The primary outcome of our analysis was the occurrence of donor-site complications, which we defined as the development of either a seroma or delayed wound healing with or without postoperative infection (Table 2). Hereinafter, we present a description of the patient characteristics and test differences in proportion and means among groups of patients using two-sample t-tests and Fisher’s exact test, respectively.

First, we used univariate logistic regression models to identify important patient characteristics and CTA measurements associated with donor-site complications and then integrated variables that presented a significant univariate association into a final multivariate logistic model in order to assess the magnitude of the association. Statistical significance was set at *p*-value < 0.05, and all statistical analyses were performed using R, version 3.6.1 (07-05-2019).

## 3. Results

Of the 90 patients included in our analysis, 27 received a bilateral reconstruction entailing the use of a total of 117 flaps. Reconstructions were carried out with 67 DIEP (74.4%) and 23 ms-TRAM flaps (25.6%). Donor-site complications occurred in 13 patients (14.4%). According to the Clavien–Dindo classification, 7 (53.8%) of them were grade I, 1 (7.7%) was grade IIIa, and 5 (38.5%) were grade IIIb (Table 2). Six (22.22%) of the 27 bilateral reconstructions in total had complications at the donor site compared to 13 (14.4%) of the 90 unilateral reconstructions in total. From the patients included in our study, two experienced total flap loss due to venous congestion. None of these patients suffered from any donor-site complications.

**Figure 3:**
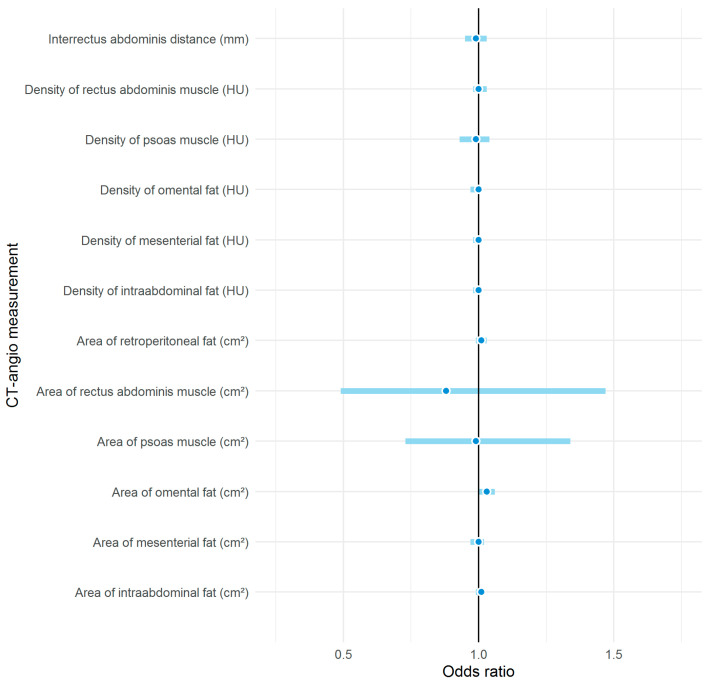
Effects of CTA measurements on postoperative complication in univariable logistic regression analyses. Effects are displayed as odds ratio (dots) together with 95% confidence interval (straight lines).

A history of abdominal surgery was present in 100% of the patients who developed a donor site complication and 55.8% of the patients who did not develop a donor site complication. When comparing the group of patients who developed donor-site complications with the group of patients who did not, the average age (55.0 years versus 54.2 years, respectively) as well as the portion of those who received chemotherapy (30.8% versus 36.4%, respectively) are similar. In all fat compartments considered, the measured area was significantly larger among obese patients than among patients of normal weight (Table 3). The area of omental fat tissue and the presence of diabetes mellitus were the two variables that showed a statistically significant association with donor-site complications in the univariate logistic regression model. In the multivariable regression model, however, only the area of omental fat tissue retained a significant association (Table 4, Figure 3) and the risk of postoperative complications increased by 3% per additional square centimetre of omental fat tissue (OR 1.03, 95% CI 1.00–1.06).

## 4. Discussion

### 4.1. Donor-Site Complications: Presumed Causes and Impact

Donor-site complications can lead to reoperations, hospitalisations, and additional outpatient treatments, resulting in a heavy burden on affected patients. As a consequence, this often comes hand in hand with a decrease in patient satisfaction and quality of life [26].

**Figure 4:**
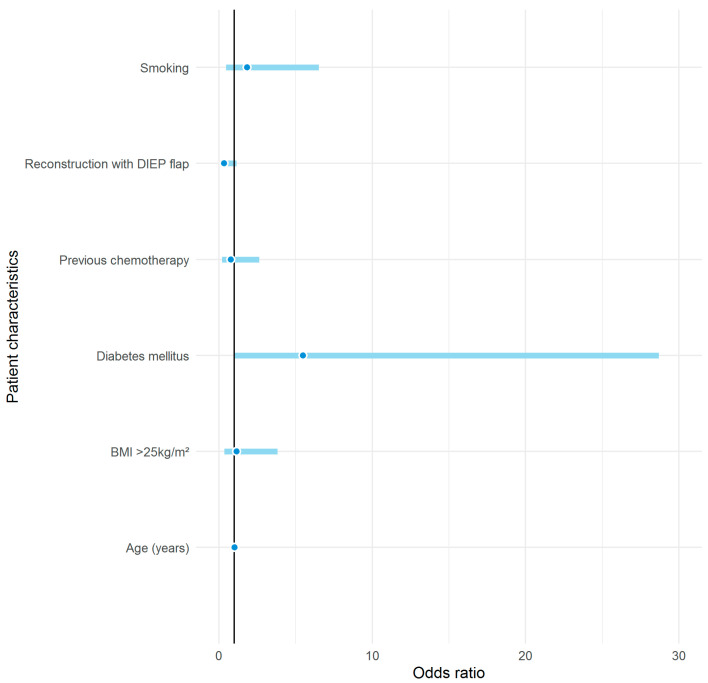
Effects of potential risk factors on postoperative complication in univariable logistic regression analyses. Effects are displayed as odds ratio (dots) together with 95% confidence interval (straight lines).

Interestingly, neither age, smoking habits, type of breast reconstruction, obesity, nor previous chemotherapy showed an increased risk for the development of donor-site complications in our analysis (Figure 4). Our results confirmed only a history of abdominal surgery as a clinical risk factor for postoperative donor-site complications; whilst the results did also indicate that pre-existing diabetes was a risk factor for donor-site complications, this association had only low statistical power. Recent studies suggest that some of these originally presumed risk factors might be obsolete, probably as a result of personalised treatment options and de-escalation of medical and surgical therapies.

Although the mean area of omental fat tissue and the areas of all other measured fat compartments were significantly smaller in patients of normal weight than in obese patients, obesity was not associated with postoperative complications. Conversely, studies in the early days of autologous breast reconstruction came to a different conclusion than our analysis [27,28]. At that time, a BMI above 30 kg/m^2^ was considered a relative contraindication to the use of abdominal tissue [29,30].

The positive association in our analysis between diabetes and donor-site complications is backed by the literature. A number of publications assert a positive association between diabetes and postoperative complications in different abdominal surgeries [31,32]. This is hardly surprising, since metabolic syndrome—a constellation of hyperglycaemia, hypertension, dyslipoproteinemia, and truncal obesity—is considered an important risk factor for morbidity and has not without reason been nicknamed “the deadly quartet” [33].

The results of our analysis regarding chemotherapy are supported by a recently published study by Beugels et al. that concludes that neoadjuvant chemotherapy is not associated with complications at the donor site [34]. Other recently published studies back this hypothesis as well [35,36,37].

Our results showed no significant difference between the age of women with donor-site complications (mean age: 55 y) and the age of women without donor-site complications (mean age: 54.2 y). Nevertheless, Hamnet et al. were able to show that older patients receive microsurgical breast reconstruction far less frequently than younger patients, as age is generally considered a risk factor for poor surgical results [38]. Yet, the literature states that a poor surgical outcome correlates more closely with a frail phenotype than it does with chronological age. As a matter of fact, numerous studies have shown that frailty, a syndrome that is characterised by the loss of biological reserves and mounting failure of homeostatic mechanisms and that manifests mainly in the elderly, is often tied to postoperative complications and longer hospital stays [38,39,40,41,42]. Therefore, we concluded that age in and of itself should not be a contraindication to autologous reconstruction and that each unique patient situation requires careful examination. By means of morphometric measurements, frailty can be quantified and by so doing the risk can be objectively assessed.

These results give ample reason to critically question the specific patient traits that have been considered established risk factors for years now and to either reaffirm them in new studies or to replace them with more precise and objectifiable ones. In any case, it might be helpful to refine the existing risk assessment with specific patient traits using additional morphometric measurements.

### 4.2. Obesity, BMI, and Areas of Fat as Predictors of Donor-Site Complications

In this retrospective cohort study, the only significant morphological risk factor for donor-site complications was the area of omental fat tissue (OR 1.03, 95% CI 1.00–1.06).

Omental fat tissue is the part of the fat tissue in the abdominal cavity that is known as the visceral or intraabdominal fat tissue. This visceral fat tissue, which manifests as truncal obesity, is a well-known risk factor for a multitude of conditions, including type 2 diabetes, cardiovascular disease, and sleep apnea, among others. In short, current evidence suggests that visceral fat is linked to all those conditions that in the past were considered positively associated with excessive total body weight per se [43].

To define and classify obesity, the BMI remains a straightforward, inexpensive, quick, and widely used tool [44]. In contrast to the BMI, however, morphological measurements taken on CT angiograms are able to describe specific areas of fat in any specific region of interest.

As previously mentioned, an extensive amount of intra-abdominal fat tissue not only increases the risk of developing a higher morbidity, it is also considered to increase the risk of sustaining a variety of other surgical complications. As such, patient-specific fat distribution is a possible predictor of postoperative complications. Indeed, this link has already been described by Kuritzkes et al. and Ding et al. in bowel resection in Crohn’s patients [16,17] and by Levi et al. in patients undergoing ventral hernia repair [45]. Furthermore, omental fat leads to truncal obesity, which in turn increases intra-abdominal pressure [46,47,48]. This increased intra-abdominal pressure is associated with hernias and reduced blood flow in the abdominal wall, causing wound healing disorders [49,50]. These findings may help explain why omental fat seems to be such an important parameter in predicting donor-site complications in our current series.

Tjeertes et al. found obesity to be a significant risk factor for wound infections whilst finding no significant link between obesity and any other donor-site complications. Better still, this particular study even suggests that obesity is negligible as an important risk factor in general surgery [51]. Studies by Modarressi et al., Chang et al., and Klasson et al. achieved similar results: no significant association between obesity and donor-site complications was discovered [52,53,54]. In opposition to these findings, the meta-analysis by Schaverien et al. determined a significant association between obesity and complications both at donor and recipient sites [12].

Some years ago, Ochoa et al. were able to prove a positive association between high BMI and wound healing disorder, though not between BMI and abdominal wall morbidity [55]. Recent studies have reassessed this view, concluding that the BMI ought not be a contraindication to abdominally-based breast reconstruction [53,56,57]. Levi et al. inferred that the area of visceral fat is a predictor of postoperative donor-site complications on par with the BMI, even surpassing it in the level of accuracy in patients with a BMI above 30 kg/m^2^ [13]. Accordingly, these results may help explain why the area of omental fat was a significant risk factor in our study and not the BMI. These results suggest that it is more accurate to use morphometric measurements, such as the area of omental fat tissue, in risk stratification for complications at the donor site than the BMI.

### 4.3. The Potential of Other Morphometric Measurements

Aside from the abdominal fat compartments, several other parameters such as the interrectus abdominis distance as well as the rectus area and density have been ascribed with predictive value to the surgical outcome, though none of these particular parameters proved significant in our analyses [14,15]. Indeed, Park et al. have exemplified the value of morphometric measurements in risk stratification of bulge/hernia formation in a recently published study by using them as part of a risk score [58].

A number of studies published throughout the last few years have demonstrated that by measuring different morphological parameters with CTA, patients at risk of various eventualities, e.g., a higher postoperative mortality in colorectal surgery or liver transplantation, can be identified in advance [59,60]. Moreover, the use of preoperative CTA-derived measurements also appears promising in the field of vascular surgery, in which a specific measurement, namely, core muscle size, correlates with postoperative mortality [61]. Nonetheless, morphological measurements of fat tissue seem to have a high impact on risk assessment for postoperative donor-site complications in general surgery [16,17,62] and in abdominally-based breast reconstruction [13].

### 4.4. Personalised Medicine

Over the past few years, personalised medicine has increasingly entered the field of surgery. Individual characteristics, including molecular make-up, physiological attributes, behavioural traits, and environmental exposures are taken into account to establish the optimal treatment for each individual patient [63]. Our results underline the importance of personalised medicine in the field of breast reconstruction by using morphometric measurements in individual risk assessment.

### 4.5. Study Limitations

This study might lack the statistical power needed to identify significant associations due to the small sample size. Therefore, the results of the presented regression model should be interpreted with caution. In addition, the limited number of complications did not allow for the investigation of possible associations between specific morphological factors and specific types of complications. What’s more, this study reflects the experience of a sole surgeon at a single institution, limiting bias, and the generalisability of our results.

On account of the retrospective design of our study, we were dependent on precise documentation of complications, and had it been a prospective pragmatic multicentre study, greater generalisability would have been achieved.

## 5. Conclusions

As the validity of previously established risk factors has been put into question, there is an increasing need for additional objective, preoperative risk assessment tools for donor-site complications after abdominally-based breast reconstruction.

Valuable information for personalised treatment in breast reconstruction surgery can be obtained through morphometric measurements without any additional costs. Based on these measurements—in the case of our analysis the measurement of the omental fat area—patients can be individually advised on the best appropriate method of breast reconstruction. This tailored treatment fully in the spirit of personalised medicine is underpinned by the aim for minimal postoperative donor-site complications and burden on patients, thus maximizing patient satisfaction and quality of life. In order to strengthen generalisability of the results, a pragmatic prospective multicentre study would be ideal.

## Figures and Tables

**Table 1:** Clavien–Dindo classification in abdominally-based breast reconstruction.

Grade	Definition	Example
I	Deviation from normal postoperative course with no need for pharmacological or surgical interventions. *	- Delayed wound healing treated with dressing changes. - Seroma evacuation by needle aspiration.
II	Pharmacological treatment required.	- Wound infection treated with antibiotics.
IIIa IIIb	Requiring surgical intervention - without general anaesthesia; or - under general anaesthesia.	- Delayed wound healing treated with vacuum-assisted closure therapy. - Seroma treated with debridement and drainage.
IV	Life threatening complication.	- Major postoperative haemorrhage.
V	Death of a patient.	- Patient died postoperatively.

* Allowed therapeutic regimens: antiemetics, antipyretics, analgesics, diuretics, electrolytes, and physiotherapy. This grade also includes wound infections opened at bedside.

**Table 2:** Donor-site complications and management classified according to Clavien–Dindo.

Clavien–Dindo	Donor Site Complication	Therapeutic Management	Number of Events
I	- Delayed wound healing	- Dressing changes	3
	- Seroma	- Needle aspiration	2
	Seroma	Observation	2
IIIa	- Delayed wound healing	- Vacuum-asissted closure therapy	1
IIIb	- Seroma	- Surgical seroma evacuation and drainage	2
	- Seroma	- Surgical debridement	2
	- Postoperative wound infection	- Sclerotherapy and surgical revision	1

**Table 3:** Total area of the different fat compartments sorted by weight status.

Variable		Normal Weight (*n* = 44)	Obese (*n* = 46)	Total	*p*-Value
Area of subcutaneous fat (cm^2^)	Mean (SD)	191.3 (53.2)	348.6 (82.9)	271.7 (105.3)	0.000
Area of intraabdominal fat (cm^2^)	Mean (SD)	26.1 (28.6)	67.1 (41.7)	47.1 (41.2)	0.000
Area of omental fat (cm^2^)	Mean (SD)	8.4 (9.1)	27.8 (24.1)	18.3 (20.7)	0.000
Area of mesenteric fat (cm^2^)	Mean (SD)	17.7 (25.7)	39.3 (26.5)	28.8 (28.1)	0.000
Area of retroperitoneal fat (cm^2^)	Mean (SD)	29.5 (22.5)	53.6 (25.3)	41.8 (26.7)	0.000

**Table 4:** Odds ratio from univariable and multivariable logistic regressions of diabetes and area of omental fat.

Variable	OR (95% CI, *p*-Value)	OR (95% CI, *p*-Value)
	Univariable	Multivariable
Diabetes mellitus	5.47 (1.04–28.77, *p* = 0.042)	4.99 (0.84–27.40, *p* = 0.062)
Area of omental fat	1.03 (1.00–1.06 *p* = 0.018)	1.03 (1.00–1.06, *p* = 0.022)
